# Improving Access to Clozapine Monitoring for Inpatient Services

**DOI:** 10.1192/bjo.2025.10443

**Published:** 2025-06-20

**Authors:** Aneal Sidhu, Miss Hollie Jones

**Affiliations:** Midlands Partnership NHS Foundation Trust, Stafford, United Kingdom

## Abstract

**Aims:** The inpatient wards at St George’s Hospital, Stafford, have a system of sending clozapine monitoring bloods (full blood count) to the local general hospital for processing.

This system is inefficient and has a significant time cost to staff. It leads to delays in getting results, both from the lab and from the clozapine monitoring service (CPMS), which can impact patient care in a number of ways.

The aim of this QI was to find out whether use of the on-site Pochi machine reduced the time it takes to get results from CPMS and simplifies the process for the wards. This machine is specifically designed for these samples and is already used by other teams.

**Methods:** QI methodology was used which highlighted a number of non value-added activities, waste and poor sustainability from the usual process.

The need for access to the Pochi machine from inpatient wards was clearly established and agreements made with the local service to use machine.

The two processes were compared by process maps and monitoring the time between blood being taken to a result from CPMS being inputted into the patient notes.

**Results:** Over a 4-week period the acute inpatient wards sent 24 blood samples to the local general hospital.

2 of these results were graded 'amber’ by CPMS meaning increased frequency of blood monitoring is needed. 1 of the 24 bloods sent in this period led to a delay in a patient’s discharge while awaiting results and 1 was sent off by a taxi to avoid delay.

Through the observation forms, the time from blood being taken to a result entered on the patient’s notes went from an average of 27 hours to 39 minutes.

The process was significantly simplified with substantial reductions in waste.

**Conclusion:** This QI has evidenced that widening access of the existing Pochi machine to all acute wards has led to a significant improvement in the time taken to obtain results from CPMS, which will benefit patients and staff.

It allows abnormal results to be acted on much quicker, improving patient safety.

It has also evidenced a reduction in non value-added activities and waste with improved environmental and financial sustainability.

